# Origin and Properties of Striatal Local Field Potential Responses to Cortical Stimulation: Temporal Regulation by Fast Inhibitory Connections

**DOI:** 10.1371/journal.pone.0028473

**Published:** 2011-12-06

**Authors:** Gregorio L. Galiñanes, Barbara Y. Braz, Mario Gustavo Murer

**Affiliations:** Neural Circuit Physiology Lab, Systems Neuroscience Group, Department of Physiology and Biophysics, University of Buenos Aires School of Medicine, Buenos Aires, Argentina; Tokyo Medical and Dental University, Japan

## Abstract

Evoked striatal field potentials are seldom used to study corticostriatal communication *in vivo* because little is known about their origin and significance. Here we show that striatal field responses evoked by stimulating the prelimbic cortex in mice are reduced by more than 90% after infusing the AMPA receptor antagonist CNQX close to the recording electrode. Moreover, the amplitude of local field responses and dPSPs recorded in striatal medium spiny neurons increase in parallel with increasing stimulating current intensity. Finally, the evoked striatal fields show several of the basic known properties of corticostriatal transmission, including paired pulse facilitation and topographical organization. As a case study, we characterized the effect of local GABA_A_ receptor blockade on striatal field and multiunitary action potential responses to prelimbic cortex stimulation. Striatal activity was recorded through a 24 channel silicon probe at about 600 µm from a microdialysis probe. Intrastriatal administration of the GABA_A_ receptor antagonist bicuculline increased by 65±7% the duration of the evoked field responses. Moreover, the associated action potential responses were markedly enhanced during bicuculline infusion. Bicuculline enhancement took place at all the striatal sites that showed a response to cortical stimulation before drug infusion, but sites showing no field response before bicuculline remained unresponsive during GABA_A_ receptor blockade. Thus, the data demonstrate that fast inhibitory connections exert a marked temporal regulation of input-output transformations within spatially delimited striatal networks responding to a cortical input. Overall, we propose that evoked striatal fields may be a useful tool to study corticostriatal synaptic connectivity in relation to behavior.

## Introduction

Synaptic transmission and plasticity are customarily studied in brain slices. Recent studies tried to fill the gap between findings in brain slices and behavior by using evoked local field responses as readout of synaptic transmission *in vivo*. For instance, in the hippocampus it has been possible to study changes in evoked local field responses in parallel with learning as well as interactions between changes in synaptic efficacy and learning *in vivo* [1], [2]. Using evoked local field potentials as a readout of corticostriatal synaptic transmission proved to be more difficult, because of concerns that they could be contaminated through volume conduction from neighboring structures. Thus, issues like the relationship between changes in the synaptic efficacy of corticostriatal connections and motor learning have been studied by correlating behavior with slice physiology findings [3].

Knowing more about the origin of local field activity in the striatum may allow tackling other currently relevant problems related to corticostriatal physiology. For instance, the degree of functional overlap between the projection fields from different cortical areas [4], [5], [6], the dependence of corticostriatal plasticity on spontaneous activity and neuromodulators [7], and the control exerted by local inhibitory networks over cortical input [8], [9] are only a few still unsolved issues. Concerning local inhibition, slice physiology studies have demonstrated that collateral inhibition and GABAergic interneurons both have synaptic influences on the medium spiny projection neurons (MSNs) of the striatum [10], [11], [12], [13]. Although *in vivo* studies have demonstrated an influence of local GABAergic networks on single MSNs [14], [15], little is known about the temporal and spatial effects of GABAergic regulation at the network level.

Here we asked whether striatal field potential responses evoked by electrical cortical stimulation are of local origin and studied their relationship with intracellularly recorded synaptic potentials and the firing activity of striatal ensembles. Moreover, we analyzed the role of fast GABAergic local connections on corticostriatal communication *in vivo* by infusing the GABA_A_ receptor antagonist bicuculline into the striatum through a microdialysis probe.

## Methods

### Ethics statement

All animal procedures performed in the present study were approved by institutional regulations of the Committee for the Care and Use of Laboratory Animals (CICUAL, Approval number RS2079/2007, University of Buenos Aires) and in accordance with government regulations of the National Food Safety and Quality Service (SENASA, Resolution number RS617/2002, Argentina). All efforts were made to minimize the number of animals used and their suffering.

### Subjects and surgery

Male CF-1 mice were housed in a colony maintained under a 12 h light: 12 h dark cycle, at constant temperature (21°–24°C) with free access to food and water. A total of 28 mice (28 to 56 days old) were used for the present study. The day of the surgery the animal was deeply anesthetized with urethane (1.2–1.5 g/kg i.p.). Long-lasting local anesthetic (bupivacaine hydrochlorate solution, 5% v/v, Durocaine, AstraZeneca S.A., Argentina) was applied subcutaneously on the scalp (0.1 ml) and the animal was affixed to a stereotaxic frame (Stoelting Co, Wood Dale, IL, USA). Body temperature was maintained at 36–37°C with a servo-controlled heating pad (Fine Science Tools, Vancouver, Canada). During the experiment, the level of anesthesia was regularly verified by testing the nociceptive hind limb withdrawal reflex and by online visual examination of the frontal cortex electrocorticogram [16]. Supplemental doses of urethane were customarily given throughout the experiment (0.3 g/kg s.c. every 2–3 h).

### Striatal field potentials

Striatal field potentials were recorded from 24 channels of a two-shank silicon probe (100 µm vertical site spacing and 500 µm horizontal shank spacing; NeuroNexus Technologies, Ann Arbor, MI). Each electrode of the silicon probe had a contact area of 413 µm^2^ and an impedance of about 0.8 MΩ. The multichannel electrode was positioned within the rostral area of the dorsal striatum with an angle of 20° in the coronal plane (0.6–1.1 mm anterior to bregma, 1.0–3.0 mm lateral to midline, 1.5–4.0 ventral to the cortical surface). Multichannel signal was referenced to a screw in the occipital bone, amplified, band-pass filtered, digitized (10 kHz) and stored in a computer for offline analysis. Signal was band pass filtered into two bands: low pass-band (5–300 Hz) and high-pass band (300–3000 Hz) to obtain, respectively, striatal local field and multiunitary action potential activity stemming from the same recording sites.

### Intracellular recordings

Intracellular recordings were performed with glass micropipettes filled with 2 M potassium acetate with impedance ranging from 60–90 MΩ. The glass electrode was lowered into the rostral portion of the dorsal striatum with an angle of 20° in the coronal plane (0.8–1.0 mm anterior to bregma, 2.0–3.0 mm lateral to midline). Microelectrodes were slowly advanced through the striatum with a hydraulic micromanipulator until a neuron was impaled (typically, 2.0–3.0 mm below cortical surface). After cell penetration and complete removal of hyperpolarizing current, we examined the stability of the recordings for 2–5 minutes before studying responses to cortical stimulation (see below).

To study the local field correlates of MSNs postsynaptic potentials a second glass electrode (2–4 MΩ) filled with 2 M NaCl was lowered into the striatum approximately 400 µm posterior to the intracellular recording site (0.6 mm anterior to bregma, 1.7 mm lateral to midline and 2.7 mm below cortical surface). The signal from the extracellular microelectrode was amplified, band-pass filtered (5–300 Hz, Lab1, Akonic) and digitized together with the intracellular signal at 10 kHz.

### Drug infusion: reverse microdialysis

To perform intrastriatal pharmacological manipulations we performed local field potential recordings with simultaneous drug infusion through reverse microdyalisis. A microdialysis probe (2 mm of exposed membrane; Bioanalytical Systems, West Lafayette) was vertically lowered (100 µm per minute) into the striatum (0–0.4 mm anterior to bregma, 1.7 mm lateral to midline, 4.0 mm below cortical surface) while being constantly perfused with artificial cerebrospinal fluid (ACSF) at a 2 µl/min rate. A precision switch with zero dead space allowed perfusing the cannula with either ACSF or ACSF containing drugs. The composition of ACSF was (in mM): 147 NaCl, 3 KCl, 0.8 MgCl, 1.2 CaCl2, 2.0 NaH2PO4, 2.0 Na2PO4; osmolarity: 290–300 mOsm/l; pH: 7.4 [14]. After baseline (ACSF) recordings, we studied the effect of the competitive glutamate AMPA receptor antagonist 6-cyano-7-nitroquinoxaline-2,3-dione (CNQX, Sigma) at 100 or 200 µM or the competitive GABAA receptor antagonist bicuculline at 100 µM (bicuculline methiodide, Fluka). Drug concentrations were chosen based on previous experience [17] and reports by others [14]. Drug effect was tested for 10 minutes and then the perfusion was switched back to ACSF. Tubing dead space and the perfusion rate were taken into account to determine the time of drug delivery to the striatum.

### Cortical electrical stimulation

In all experiments, a concentric bipolar electrode (SNE-100, Better Hospital Equipment, New York, NY; outer contact diameter 0.25 mm, central contact diameter 0.1 mm, contacts separation 0.75 mm, contact exposure 0.25 mm) was placed into the prelimbic area of the medial prefrontal cortex (1.7–2.1 mm anterior to bregma, 0.4 lateral to midline, 2.0 mm ventral to the cortical surface, ipsilateral to the striatal recording hemisphere) according to Franklin and Paxinos [18]. Constant current pulses (0.3 ms duration at 0.1 Hz, 100–700 µA; Iso-Flex and Master 8, AMPI, Jerusalem, Israel) were applied to study corticostriatal synaptic connectivity through evoked striatal field potentials and postsynaptic potentials.

### Histology

At the end of each experiment, animals received a lethal dose of urethane and were transcardially perfused with 10 ml cold saline solution and 20 ml of paraformaldehyde (4% w/v) in 0.1 M phosphate-buffer (PB). Brains were removed, immersed for 30–45 minutes in the same fixative at room temperature, and stored in 0.1 M PB containing 15% sucrose at 4°C for 24–72 hours. Coronal brain sections were cut with a freezing microtome (50 µm) for histological reconstructions.

Location of the cortical stimulation electrode and the microdialysis probe was assessed by visual examination of the mechanical tissue damage in the coronal sections using a transmitted light microscope at low magnification. In order to determine the location of the striatal recording sites, before each electrophysiological experiment the multi-electrode was immersed in a red fluorescent dye 1,1′-dioctadecyl-3,3,3′,3′-tetramethylindocarbocyanine perchlorate (100 mg/ml in acetone; DiI, Molecular Probes) and air dried for 30 minutes before use. This allowed detecting the fluorescent material deposited in the tissue with an epifluorescence microscope. In all cases, sections of interest were microphotographed for subsequent reconstruction of the final recording, stimulation and dialysis sites.

### Data Analysis

Striatal field potentials were off line analyzed with custom made Matlab routines. Electrical stimulation of the mPFC evoked a complex striatal field potential which typically consisted of a positive-negative-positive (P1-N2-P2) waveform. An earlier negative component (N1) described by others [19], [20], [21], [22] was not readily detected in our experimental settings, probably masked by the stimulation artifact.

**Figure 1:**
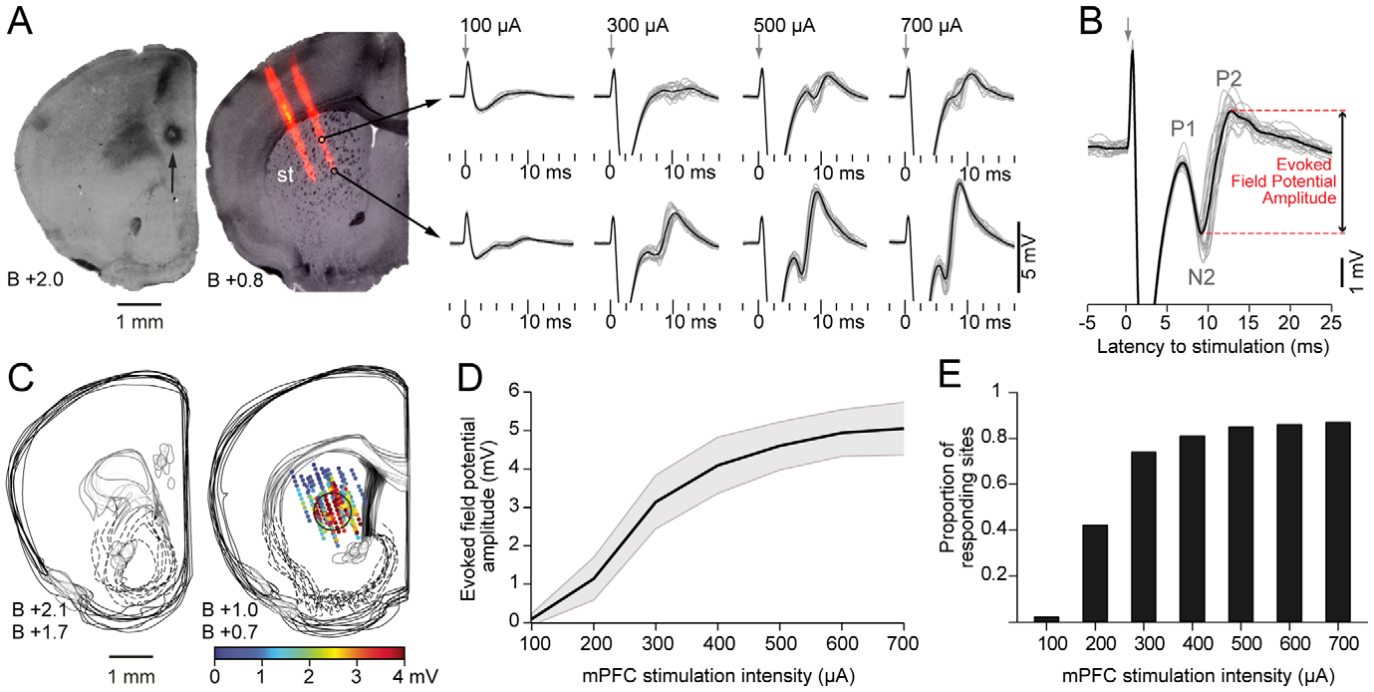
Evoked field potential amplitude changes along the dorsal striatum and with stimulation intensity. A. Representative histological sections showing the location of cortical (arrow, *left*) stimulation electrode and striatal (st, *right*) recording electrode. The striatal image was composed by overlaying microphotographs of the same section under transmitted light (tissue) and epifluorescence (electrode, red). Multichannel silicon probes were immersed in a DiI solution before electrophysiological experiments. Traces on the right are local field potentials evoked by stimulating the cortex with different current intensities (individual trials in gray, average in black). Note the higher amplitude of evoked responses at higher stimulation currents. B. Detail of a representative evoked field response. Field potential amplitude was measured between the N2 and P2 peak for each individual trial and then averaged. In these and all further traces positive is upward. Time 0 corresponds to cortical stimulation. C. Topographical reconstruction of stimulation sites (*left*) and striatal evoked responses (*right*) in 12 experiments. Focal stimulation (300 µA) at the prelimbic area produces a maximal response in a restricted region of the dorsal striatum conforming a “hot spot” (circle). D. Amplitude of the striatal field response at the hot spot as a function of stimulation current intensity (n = 12 experiments, mean±SD). E. The number of striatal sites that respond to prelimbic cortex stimulation (evoked field potential amplitude higher than 0.3 mV) increases with stimulation intensity. However, many recording sites (38 out of 288 recorded sites) remained unresponsive even at 700 µA.

The amplitude of the evoked local field potentials was determined as the voltage difference between the N2 peak and the subsequent P2 peak (Figure 1B). To measure the amplitude of the evoked potentials, the recorded signal was denoised using a zero-phase digital filter (8 pole butterworth filter, low-pass cutoff 300 Hz). For every individual trial the voltage and latency of the N2 and P2 peak were semiautomatically determined by respectively detecting the local minimum and maximum of the evoked field potential. For quantitative time-course population analysis, the averaged responses of 5 minutes of recording were used. When CNQX infusion abolished evoked field potential responses and the N2 and P2 peaks were not readily detected, measurements were taken at the timestamps determined during the immediately previous baseline condition for the same recording site. Under some experimental conditions, additional field responses (secondary, tertiary, etc) were detected after the main evoked field response. The amplitude of the secondary field response was measured in the same way as the main field response. Bicuculline infusion typically enhanced or induced secondary waves. For recording sites that did not display a secondary field response during the baseline condition, the amplitude was measured at the timestamps determined for the bicuculline condition. Therefore, in the absence of a true secondary field response during baseline recordings the measurement yielded, in occasions, negative values.

Due to the relatively low impedance of recording electrodes used (∼0.8 MΩ), action potential evoked responses were multiunitary. To quantitatively study multiunitary responses, high-pass signal was rectified, smoothed with a 1 sample-sliding window (30-samples centered-average), and averaged across 30 trials. The standard deviation for the 30 trials was computed for 100 ms of the high-pass signal preceding the stimulation onset. The rectified and smoothed high-pass signal which surpassed 3 times the standard deviation was considered as a significant response.

Intracellular recordings were analyzed with Clampfit 10 (AxonLabs). Statistical analysis was performed with Statistica 7.0. Repeated measures ANOVA and Student's paired t test was applied when the data distributions fulfilled parametric assumptions. Otherwise the non-parametric Wilcoxon paired test was applied for comparing before and after drug effect.

## Results

### Prelimbic cortical stimulation induces a topographically organized and stimulation intensity dependent striatal field response

It is known that corticostriatal projections are topographically organized with the medial prefrontal cortex (mPFC) projecting to the medial part of the dorsal striatum and nucleus accumbens [23]. To study the physiological correlate of such organization we performed *in vivo* multi-site simultaneous recordings of striatal field potentials evoked by electrical stimulation at the prelimbic region of the mPFC (Figure 1A). Typical striatal responses consisted of a positive-negative-positive wave with a negative peak (N2) occurring 8.4±1 ms and a positive peak (P2) at 12.3±1.2 ms after mPFC stimulation (n = 10, mean±SD, Figure 1B). An early N1 wave reported by others in striatal slice recordings [19], [21], [24] was probably hidden by the stimulation artifact in our experimental preparation. In some instances, an additional negative wave (N3) smaller in amplitude was observed after the P2 peak of the main field response (see below). Similar shapes of evoked field potentials recorded *in vivo* have been reported for other unlayered structures, such as the subthalamic nucleus and basolateral amygdala [25], [26]. Overall, our findings are consistent with previous *in vivo* and *in vitro* studies on evoked striatal field potentials [19], [21], [22], [24], [27].

The amplitude of the striatal evoked potentials varied with the position of the recording site within the striatum. Field responses evoked from stimulation sites located within the prelimbic area were maximal in the centromedial region of the dorsal striatum and decayed towards the dorsal and lateral striatum, conforming a “hot spot” (Figure 1C). This pattern of regional response is consistent with the anatomy of the corticostriatal projection from the mPFC [23] suggesting that evoked field potentials are a physiological correlate of such connections.

As expected, the amplitude of the striatal field potentials increased with mPFC stimulation intensity (Figure 1D). Although most of the recorded sites were unresponsive to the lowest stimulation intensity (100 µA), a small fraction of the recorded sites located at the maximally responsive area of the striatum did show a small but consistent field response (Figure 1A, lower traces). Higher stimulation intensities (200–500 µA) recruited a larger number of responsive sites with a corresponding increase in the amplitude of the evoked potentials (Figure 1 D and E). The input-output curve shows that striatal evoked responses tend to reach a “plateau” at intensities higher than 500 µA, suggesting a saturation of the corticostriatal connections influenced by the stimulating electrode and highlighting the physiological nature of the recorded potentials (Figure 1D, *p*>0.31 for 500 vs 600 µA and *p*>0.9 for 600 vs 700 µA, Tukey post-hoc test, repeated measures ANOVA). Recording sites located far from the hot spot were unresponsive even at 700 µA, indicating a restricted corticostriatal connectivity map originated at the prelimbic region of the mPFC.

### Striatal field responses increase in parallel with dPSP amplitude in medium spiny neurons

**Figure 2:**
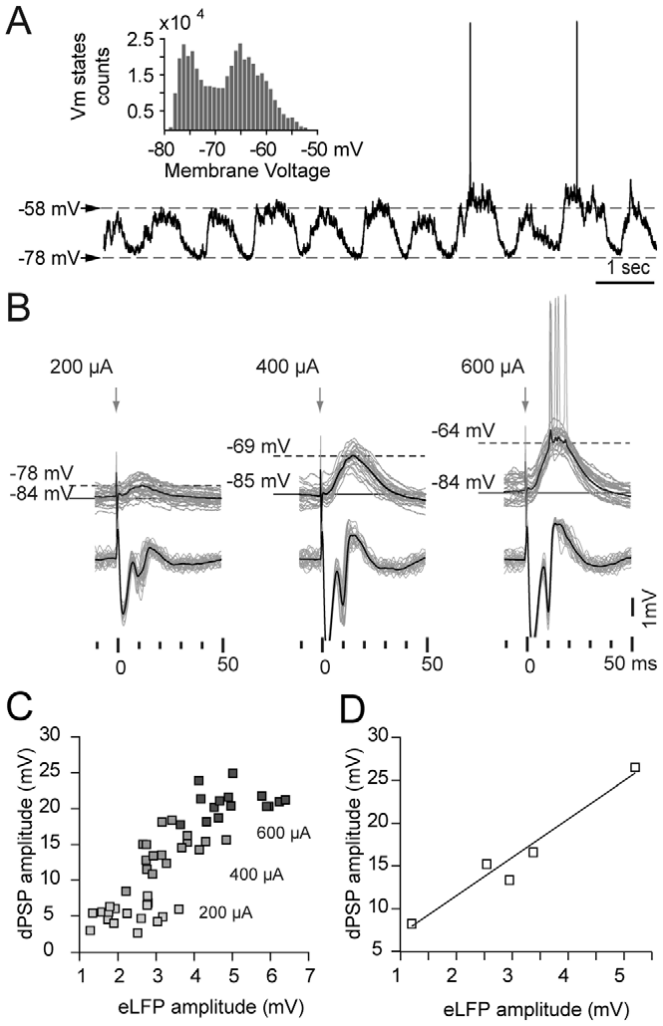
Striatal field responses are related to dPSPs in medium spiny neurons. **A.** As in rats, the membrane potential of mouse MSNs alternates between up and down states. The histogram (1 mV bin) shows a biphasic distribution of Vm values corresponding to 20 seconds of the illustrated intracellular recording. **B.** Simultaneous intracellular (*above*) and local field (*below*) recordings obtained with separate glass microelectrodes after stimulating the prelimbic cortex with different current intensities. Individual traces are displayed in gray, averages in black. The through in the local field response coincides temporally with the peak of the dPSP. **C.** The amplitudes of simultaneously recorded striatal dPSPs and evoked local field potentials (eLFP) increase in parallel with increasing stimulation intensities. Data corresponds to one simultaneously recorded pair stimulated at 200, 400 and 600 µA. **D.** Correlation between eLFP and dPSP amplitude evoked by prelimbic cortex stimulation at 400 µA in five different experiments (n = 5, r^2^ = 0.94, *p*<0.01).

To assess whether striatal local field potentials are related to synaptic activity in MSNs we performed simultaneous intracellular recordings of MSNs and striatal field potentials. As in rats, striatal MSNs displayed a bistable membrane potential, which alternated between a very hyperpolarized “down” state and a more depolarized “up” state (−82±2 and −65±3 mV, n = 7, mean±SD; Figure 2A). Action potential discharge probability was very low and restricted to the “up” states, as expected for MSNs, and input resistance measured at the down state was also similar to that previously reported in rats (38.7±4.5 MΩ, n = 4, mean±SD, [28], [29]).

Upon prelimbic cortex stimulation, MSNs showed a depolarizing postsynaptic potential (dPSP) which peaked at 11.8±2.4 ms (n = 7, mean±SD), with an amplitude that increased with stimulation intensity (Figure 2B). Although in some instances dPSPs were strong enough to elicit action potentials, this was seldom the case for stimulation intensities below 500 µA. Simultaneously recorded field potentials revealed a temporal correspondence of the N2 component of the field potentials and the peak of the dPSPs (N2 latency 10.8±1.1, n = 7 mean±SD, *p*>0.3 compared to dPSP peak time, Student's paired t test). Furthermore, increasing stimulation intensities led to a parallel increase of the dPSP and striatal field potential amplitudes (Figure 2C).

**Figure 3:**
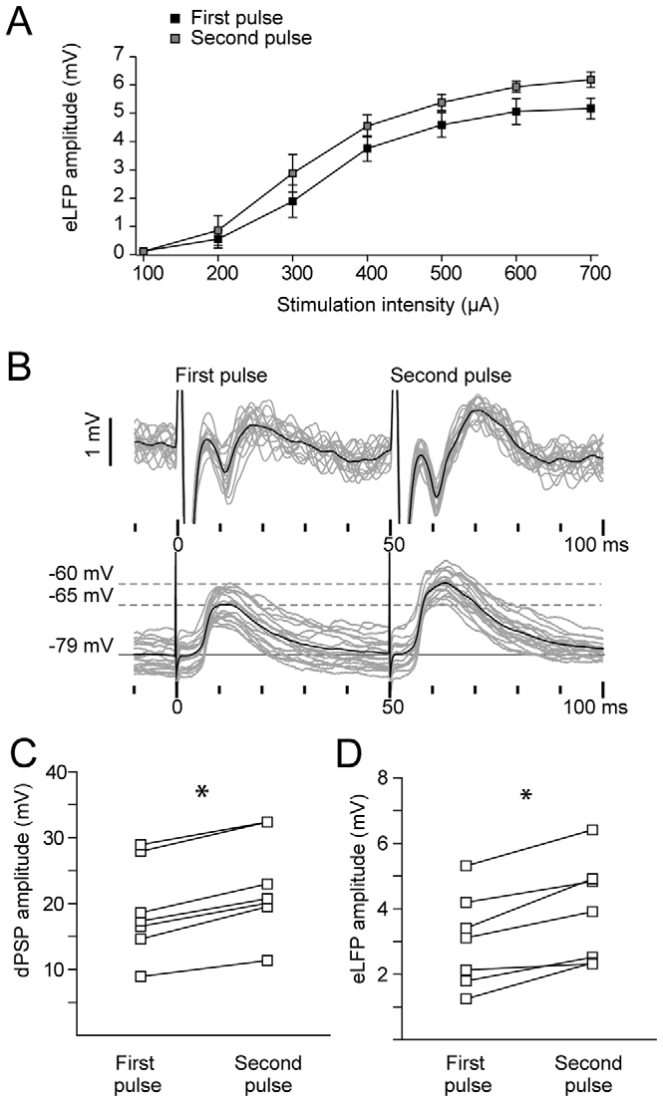
Evoked striatal field potentials show paired pulse facilitation. **A.** Amplitude of the striatal field response (mean±SEM, n = 5) as a function of stimulation current intensity for cortical paired pulse stimulation (interstimulus interval 50 ms). **B.** Simultaneous recordings of striatal eLFP through a glass micropipette (*above*) and MSN membrane potential (*below*) after cortical paired pulse stimulation. **C–D.** Paired pulse stimulation at the prelimbic cortex (400 µA and 50 ms interstimulus interval) induces a facilitation of the response to the second stimulus in MSNs (C, *p<0.0001, Student's paired t test, n = 7) and evoked striatal field potentials (D, *p<0.005, Student's paired t test, n = 7).

Another observation that supports the relationship between evoked field potentials and membrane potential changes in MSNs is that striatal field responses show paired pulse facilitation. Corticostriatal paired pulse facilitation is a well known form of short-term synaptic plasticity that has been extensively studied *in vitro* as a tool for interpreting presynaptic changes associated with long term plasticity [30], [31], [32]. After paired pulse stimulation *in vivo* (50 ms interstimulus interval), the second striatal evoked potential was consistently higher than the first one for cortical stimulation intensities ranging from 200 to 700 µA (Figure 3A). As expected, similar results were obtained for the amplitude of MSNs dPSPs (23±7% facilitation at 400 µA stimulation intensity, mean±SD, Figure 3B–C). Simultaneously recorded striatal field potentials showed an increase of 32±9% (mean±SD, Figure 3D).

Altogether, the results obtained so far suggest that striatal field responses are related to MSN synaptic activity.

### The amplitude of evoked striatal fields is related to local multiunit activity

**Figure 4:**
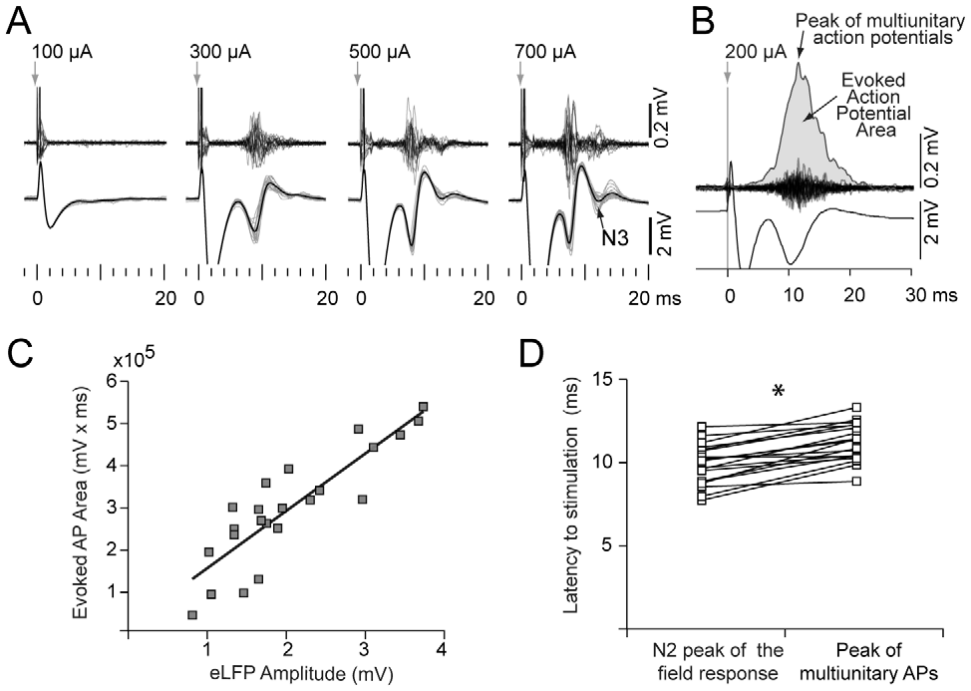
Evoked field response precedes the peak of multiunitary action potential response. **A.** Field (3–300 Hz) and action potential (300–3000 Hz) responses at the hot spot to increasing stimulation current intensities. Note the secondary field response (N3 peak, black arrow) at high stimulation intensities (20 trials at each intensity). **B.** To quantify the action potential response, trials were rectified, smoothed and averaged, allowing the computation of the area and peak latency of the multiunitary action potential response. **C.** Correlation between the amplitude of the evoked field potential and the area of the action potentials response at 300 µA; each point stems from one recording site at the hot spot from 23 different experiments. r^2^ = 0.84, *p*<0.0001. **D.** Field response N2 peak precedes the peak of multiunitary action potential response (n = 23, * *p*<0.005, Student's paired t test).

Next, we investigated whether the local field responses were correlated to the striatal firing. To this end, striatal signal was band-pass filtered (see Methods) to obtain local field potentials and multiunitary action potentials stemming from the same recording sites. Due to the low impedance and relatively large contact area of the recording electrodes, it was not possible to systematically identify single unit action potentials evoked by cortical stimulation. Nevertheless, multiunitary action potentials were readily observed in those striatal recording sites that displayed field evoked responses (Figure 4A–B). Note that, however, recorded spikes are probably contributed by a minority of the neurones receiving inputs from the prelimbic cortex, as intracellular recordings show that most MSN show subthreshold responses without action potential discharges even at a stimulation intensity of 400 µA (Figure 2). As expected, higher stimulation intensities induced stronger multiunitary responses, which were also strongly correlated with evoked field potential amplitudes (Figure 4C).

As mentioned before, in some recording sites it was possible to obtain a second smaller wave (N3) mounted on the main field response (Figure 4A). Such secondary field response was more likely to appear if the recording site was within the “hot spot” and the stimulation intensity applied was high. Noticeable, this wave was temporally associated to a second burst of multiunitary action potential activity (Figure 4A, 700 µA), supporting the relation between the evoked field potential and striatal action potentials. However, when comparing the latencies of the local field and action potential responses, it seemed that striatal field responses took place prior to the action potentials. In fact, the N2 component of the evoked potentials preceded the peak of multiunitary action potential activity (Δt: 1.31±0.78 ms, n = 23, mean±SD, paired t test: *p*<0.001, Figure 4D).

Therefore, the data suggest that cortically evoked field potentials reflect subthreshold activity which, as expected, is correlated to striatal spiking activity.

### Local origin of evoked striatal field potentials

**Figure 5:**
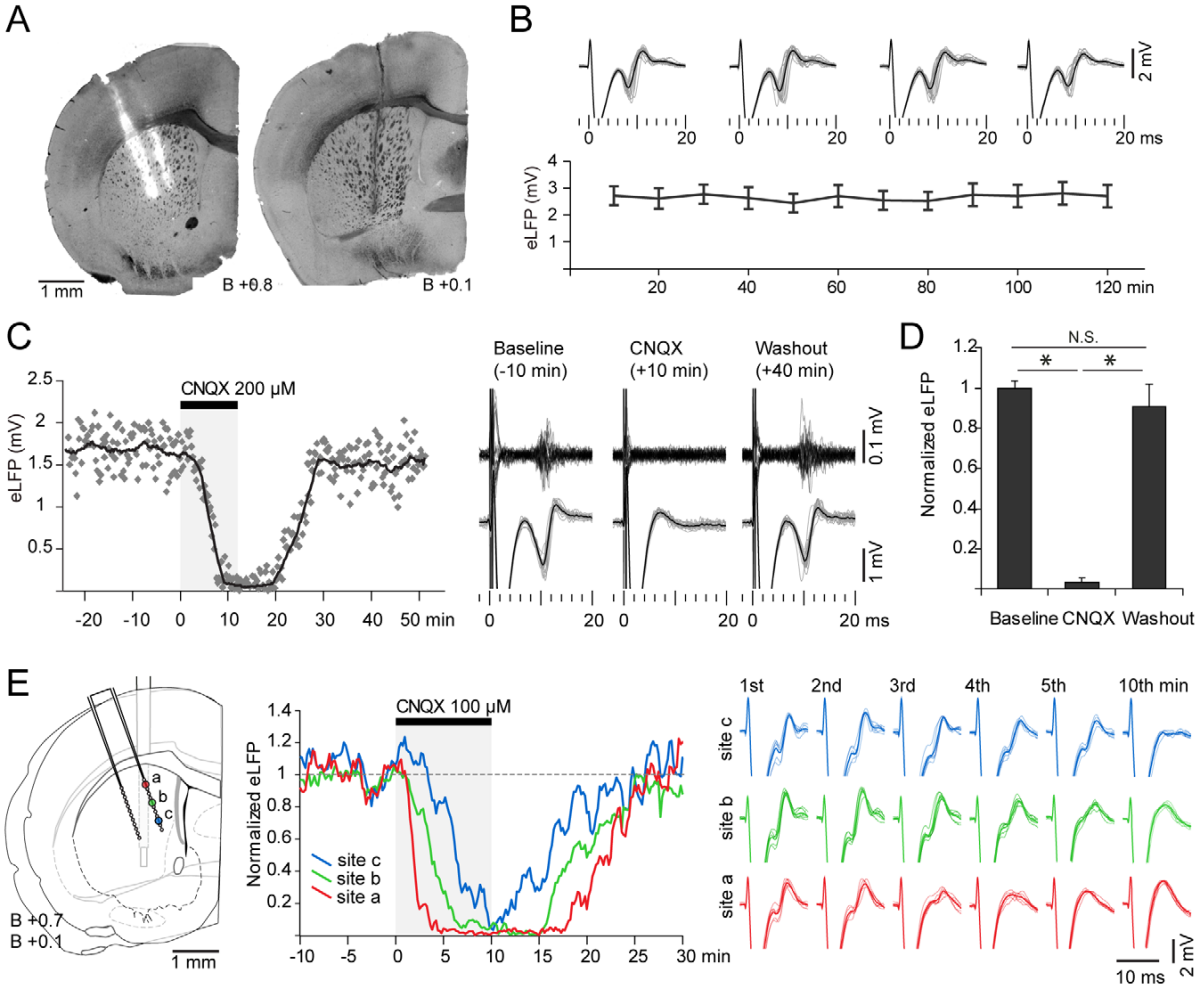
Local administration of CNQX blocks synaptic responses in the striatum. **A.** Histological sections showing the location of the multichannel electrode (left) and microdialysis probe (right) in a representative experiment. **B.** Striatal field responses evoked by stimulating the prelimbic cortex remained stable for hours under continuous infusion of ACSF through the microdyalisis probe (n = 3). **C.** *Left:* Time course of the blocking effect of CNQX on striatal field responses evoked by cortical stimulation in a representative experiment. Each point is the amplitude of a single evoked response, which was evaluated every ten seconds during the experiment. The superimposed line corresponds to the smoothed data with a moving average window (20 samples). *Right:* Field potential responses corresponding to the individual trials depicted in gray, and multiunit activity recorded from the same electrode contact, before, after 10 minutes of CNQX infusion, and after 40 minutes of washing out with ACSF. Data are from the experiment shown at the left. **D.** CNQX administration (200 µM) almost completely blocked the striatal field response to cortical stimulation (* *p*<0.001, Tukey post-hoc test, repeated measures ANOVA, N = 4). **E.** *Left:* Histological reconstruction showing the location of three recording sites in the multichannel electrode (*a, b, c*) and the microdialysis probe in one of the CNQX experiments. *Middle:* Time course of CNQX effect at sites *a, b* and *c*. Note that CNQX reached first the closest recording site *a* than the more distant sites *b* and *c*. Distance between illustrated recording sites: 400 µm. *Right:* Striatal field response during the first 5 minutes and at the 10th minute of CNQX infusion in the same experiment, showing that by the fourth minute, response in site *a* was abolished but remained almost unchanged 800 µm away in site *c*. Note that the time course of CNQX effect depends on the location of the recording electrode and not on the basal amplitude of the response (compare site *a* with site *c*).

As there is no intrinsic source of excitation in the striatal circuit, when AMPA receptors are blocked, synaptic activity disappears in striatal neurons in corticostriatal organotypic cultures [33]. In order to unequivocally determine whether striatal evoked field potentials are synaptically originated, we studied the effect of intrastriatal microinfusion of the AMPA receptor antagonist CNQX by means of reverse microdialysis. Microdialysis probes were positioned within the striatum between 400 and 800 µm posterior to the multichannel recording electrode (Figure 5A). In agreement with previous reports showing that the microdialysis procedure does not alter basic physiological properties of MSNs *per se* [14], [17] we found that cortically evoked striatal field potentials displayed waveforms, amplitudes and latencies comparable to those obtained without a microdialysis cannula. Furthermore, striatal evoked potentials remained unchanged after 2 hours of ACSF infusion (Figure 5B).

A complete blockade of the evoked field potentials was typically obtained within 10 minutes of CNQX infusion (Figure 5C–D). Similar results were obtained for drug concentrations of 100 and 200 µM (n = 3 and 4 experiments, respectively), revealing the glutamatergic synaptic nature of the evoked field potentials. Importantly, after washing out with ACSF, evoked field potentials completely recovered to basal amplitudes (Figure 5C–D).

The latency and magnitude of the CNQX effect depended on the distance between the recording site and the dialysis probe. Recording sites located closer to the dialysis probe displayed a faster and more potent blockade than the farther ones. For instance, in the experiment depicted in figure 5E, the evoked response in the recording site *a* was almost abolished by the 4th minute of CNQX infusion, whereas the response at the recording site *c* was only diminished by a 20%. This evidence demonstrates not only that striatal field responses are locally and synaptically generated but also that field responses recorded from different electrodes within the striatum are locally generated as well and do not reflect volume conduction from an intrastriatal current source.

### Local GABA networks modulate local field responses to prelimbic cortex stimulation

Although the effect of local GABAergic connections on the excitability of MSNs has been investigated *in vivo* [14], [15], [34], its effects at the network level have only been investigated *in vitro* and by means of computer simulations (see for example [8], [35]). Here we asked whether cortically evoked striatal field potentials are influenced by local GABAergic neurotransmission by means of intrastriatal infusion of 100 µM bicuculline through reverse microdialysis.

**Figure 6:**
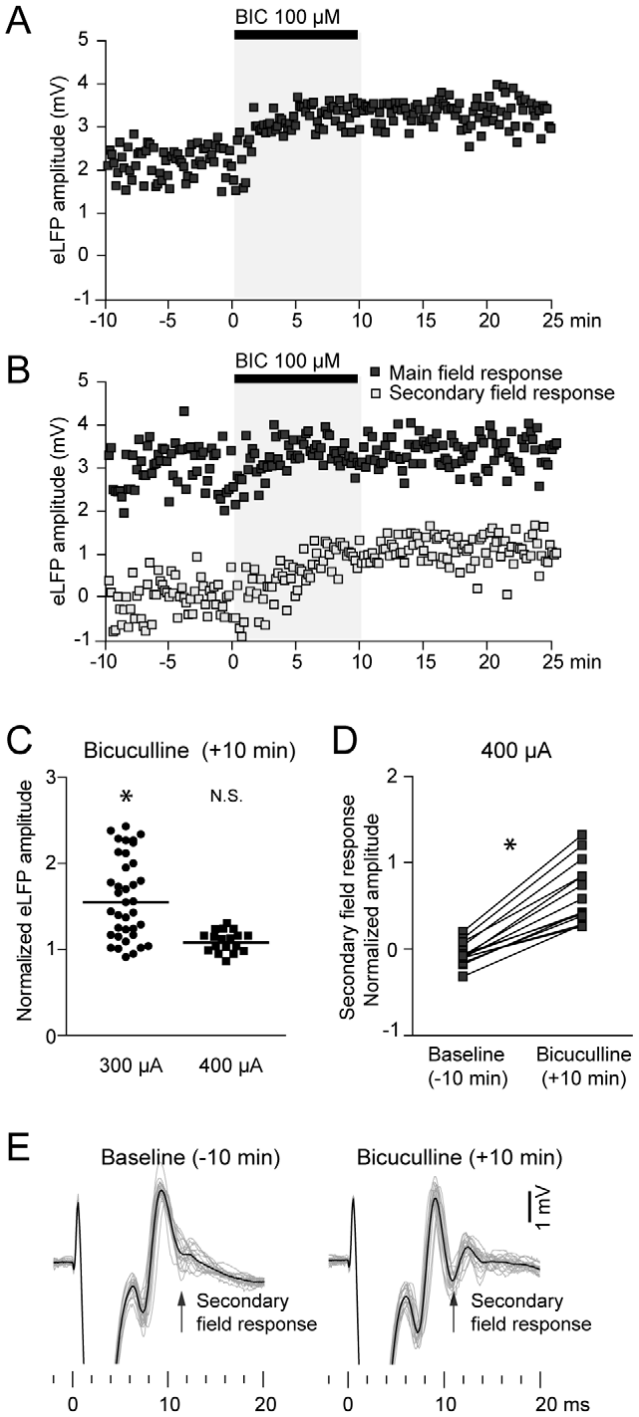
Local administration of a GABA_A_ receptor antagonist increases striatal evoked field potential responses. **A.** Time course of the evoked field potential amplitude of a representative striatal site tested with 300 µA stimulation intensity. Note a ∼50% amplitude increase after 10 minutes of bicuculline infusion. **B.** Time course of the evoked field potential amplitude of a representative striatal site tested with 400 µA stimulation intensity. Note that the amplitude of the main field response does not signifcantly change after bicuculline infusion. However, a 1 mV secondary field response (open squares) becomes apparent (see traces shown in E). **C.** Population data of the main field response amplitude after 10 minutes of bicuculline for 300 and 400 µA stimulation intensity. Values are normalized to baseline. * *p*<0.0001,Wilcoxon paired test. **D.** Bicuculline consistently increased or induced the appearance of a secondary field response in all recording sites stimulated at 400 µA that showed a field response during baseline condition (32±5%, * *p*<0.001 Wilcoxon paired test). Values are normalized to the main field amplitude during baseline. **E.** Representative traces showing the effect of intrastriatal bicuculline infusion by reverse microdialysis on local field responses to prelimbic cortex stimulation at 400 µA. Note the increased secondary field response after bicuculline infusion.

We performed two sets of experiments using 300 (4 mice) or 400 µA (3 mice) cortical stimulation intensity. When stimulating the prelimbic cortex at 300 µA, intrastriatal infusion of bicuculline increased field potential responses by 34±6% (*p*<0.0001 Wilcoxon paired test, Figure 6A,C). Unexpectedly, when stimulating at a higher current intensity (400 µA) we did not find a significant increase of the evoked field potential amplitude (*p*>0.6 Wilcoxon paired test, Figure 6B–C). In addition, bicuculline tipically induced the appearance of a secondary field response following the main striatal evoked potential (Figure 6B,D–E), suggesting a temporal expansion of the overall response to cortical commands. Interestingly, similar results have been reported *in vitro* by using picrotoxin instead of bicuculline [36]).

**Figure 7:**
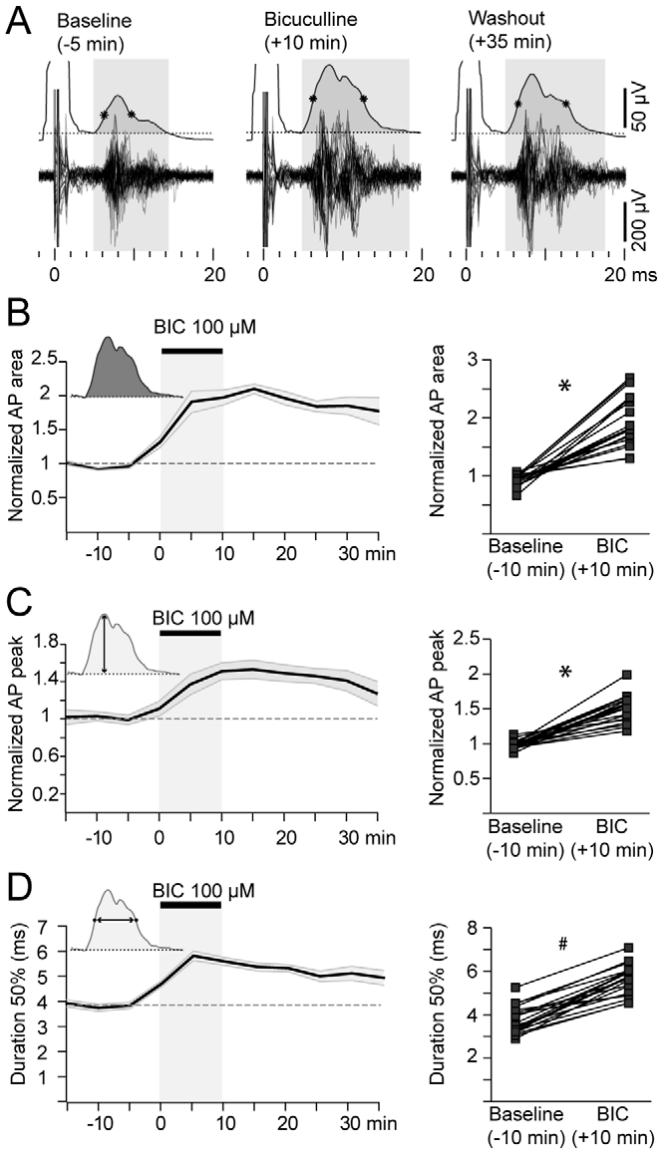
Intrastriatal bicuculline increases the amplitude and duration of striatal output. **A.** Representative multiunitary response to cortical stimulation before, during and after delivering bicuculline into the striatum. The top trace corresponds to the rectified, smoothed and averaged action potential activity of 20 individual trials. Dotted line reflects 3 SD of multiunitary activity during 100 ms prior to stimulation onset (400 µA). Note the increase in the amplitude and duration of the response after bicuculline. Similar results were obtained for 300 µA cortical stimulation. **B–D.** Effect of bicuculline on the area (**B**), amplitude (**C**) and duration (**D**) of multiunitary action potential responses of 20 recording sites at the hot spot from 3 different experiments. * *p*<0.0001, Wilcoxon paired test. # *p*<0.0001 Student's paired t test.

Regardless of the stimulation intensity, the multiunitary action potential response was increased after bicuculline infusion (Figure 7A). The action potential area increased by 2 fold (197±11% compared to baseline, *p*<0.0001, Wilcoxon paired test, Figure 7B). This change was due to an increase in its peak amplitude (50±11% compared to baseline, *p*<0.0001, Wilcoxon paired test, Figure 7C) and duration (from 3.84±0.56 ms during baseline to 5.59±0.69 ms after 10 minutes of bicuculline; mean±SD, *p*<0.0001, Student's paired *t* test, Figure 7D), indicating that striatal output is strongly modulated by local GABA.

The dissociated effect of bicuculline on the amplitude of striatal field and action potential responses at 400 µA cortical stimulation supports the notion of a mainly subthreshold origin of the evoked field responses.

**Figure 8:**
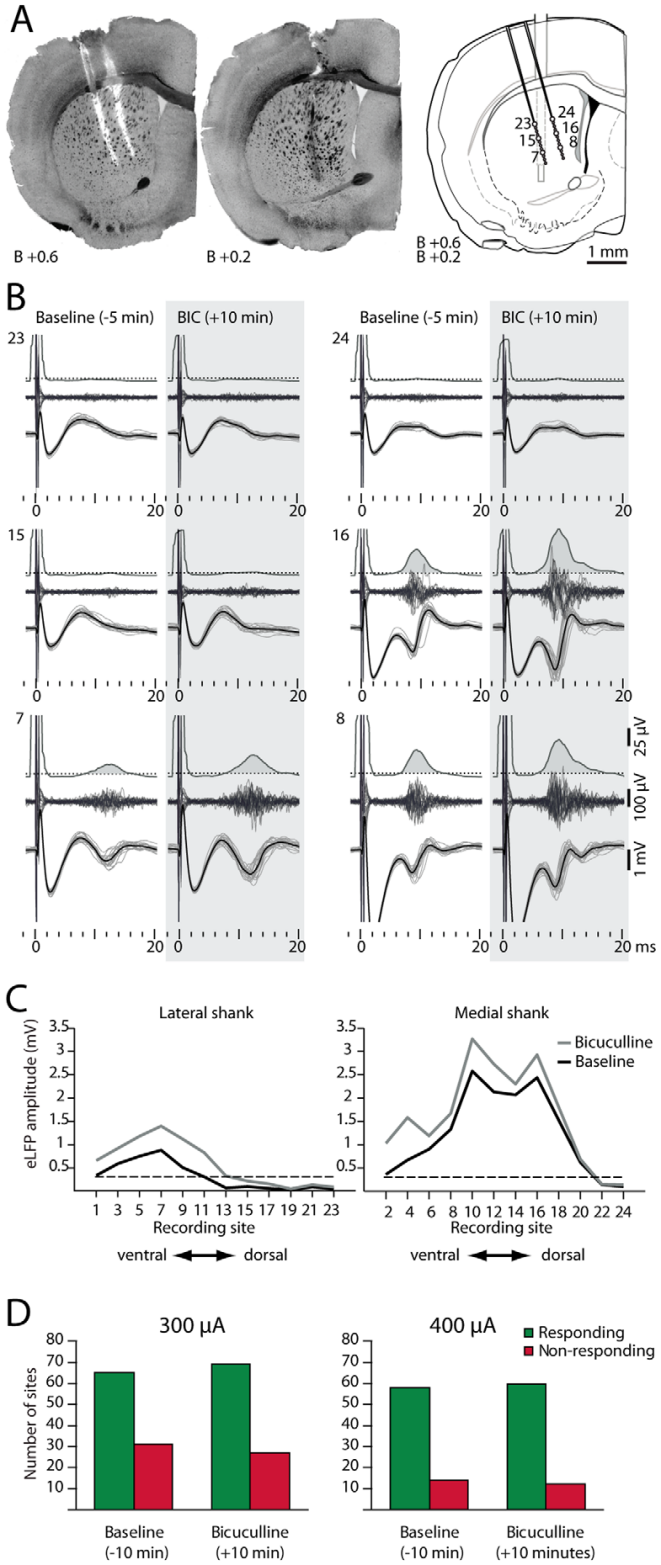
Acute blockade of striatal GABA_A_ receptors does not expand functional corticostriatal maps. **A.** Histological reconstruction of the recording electrode and the microdialysis probe in the striatum for a representative experiment. **B.** Signal recorded from six striatal sites (7, 8, 15, 16, 23 and 24) before and after bicuculline corresponding to the recording sites depicted in A after 300 µA prelimbic stimulation. Note that evoked field potentials recorded from sites 7, 8 and 16 were increased after bicuculline infusion, whereas sites 15, 23 and 24, which were unresponsive under ACSF, remained unresponsive to prelimbic stimulation after 10 minutes of bicuculline infusion. **C.** Evoked local field potentials of 24 recording sites from the experiment shown in A and B. Dashed line corresponds to the threshold of significant field potential response (>0.3 mV). **D.** Proportion of responding sites before (baseline) and after 10 minutes of bicuculline infusion for 300 or 400 µA cortical stimulation. Data are pooled from 4 and 3 experiments, respectively. Bicuculline administration did not significantly change the proportion of responding versus non-responding sites for either 300 µA (*p*>0.3 Fisher Exact Probability one-tailed test, 96 sites) or 400 µA (*p*>0.4 Fisher Exact Probability one-tailed test, 72 sites).

To determine whether the extension of corticostriatal synaptic maps is limited by GABA neurotransmission, we computed the number of responding sites (sites displaying a field potential amplitude >0.3 mV) before and after bicuculline administration (Figure 8C–D). Both, low and high cortical stimulation intensity yielded similar results: at 300 µA only 4 out of 31 non-responding sites became responsive after bicuculline infusion whereas the proportion for 400 µA experiments was 2 out of 14 (Figure 8D). These results indicate that the extent of the synaptic corticostriatal map revealed by cortical stimulation does not appear to be shaped by GABA neurotransmission.

### Paired pulse facilitation is still expressed after local GABA_A_ receptor blockade

Studies in slices indicate that corticostriatal paired pulse facilitation is mainly presynaptic in nature [30], [31], [37] (although see [32]). However, *in vivo*, local GABA networks made up by fast spiking interneurons and axon collaterals of MSNs may be recruited preferentially by the first or second stimulation pulses, and then have an influence on paired pulse facilitation of cortically evoked fields [11], [12], [15].

**Figure 9:**
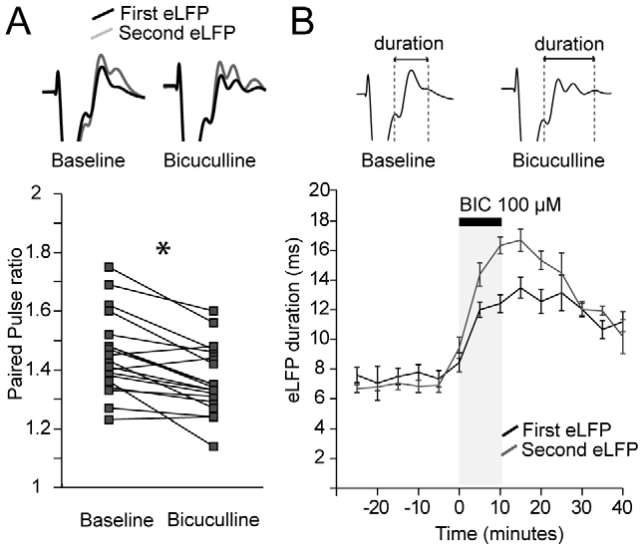
Intrastriatal bicuculline infusion does not block paired pulse facilitation and enhances field response duration. **A.** Paired pulse ratio (interstimulus interval 50 ms) of the amplitude of the evoked field potentials during baseline and after bicuculline. Top traces: superimposed average traces of the first and second evoked field potentials during baseline and bicuculline condition. Paired pulse facilitation was not blocked by bicuculline indicating that paired pulse facilitation is not due to differential effects of GABAergic neurotransmission during the first and second stimulation pulse. N = 3 experiments. * *p*<0.05 Student's paired t test. **B.** The overall duration of the striatal field potentials was determined as the time between the first positive peak P1 of the field response and the positive peak of the last supplementary response. During baseline condition, the duration of the first and second evoked potential was comparable. After 10 minutes of bicuculline infusion the overall duration of the first evoked potential was increased 65±7% whereas the second was increased 146±9%.

Before bicuculline administration, prelimbic cortex stimulation induced a strong paired pulse facilitation of the striatal field response (45±3%, mean±S.E.M.). After 10 minutes of GABA_A_ receptor blockade, paired pulse facilitation was still present (36±1%, mean±S.E.M., n = 3 experiments at 400 µA; Figure 9A), indicating that paired pulse facilitation cannot be completely explained by a differential recruitment of GABAergic circuits during the first and second stimulation pulses.

Consistent with the effect of bicuculline on the striatal multiunitary response, we found that the overall duration of the striatal field response to the first cortical pulse was increased by 65±7% after 10 minutes of bicuculline infusion (mean±S.E.M., *p*<0.0005, Tukey post-hoc test, repeated measures ANOVA, Figure 9B), whereas the response to the second pulse increased 146±9% (mean±S.E.M., *p*<0.0005, Tukey post-hoc test, repeated measures ANOVA, Figure 9B). Interestingly, the duration of the field responses to the first and second pulses were comparable during ACSF infusion (*p*>0.1, Tukey post-hoc test, repeated measures ANOVA), but the response to the second pulse was significantly longer than that to the first pulse after 10 minutes of bicuculline infusion (*p*<0.0005, Tukey post-hoc test, repeated measures ANOVA). These results confirm the notion that local GABAergic neurotransmission plays a crucial role in the temporal processing of cortical commands and indicate that recent inputs may influence the upcoming GABAergic regulation of the striatal network.

## Discussion

In the present study we show that cortically evoked striatal field potentials are synaptically and locally generated. The physiological significance of the evoked potentials was revealed by simultaneous intracellular recordings and pharmacological manipulations which indicate that striatal evoked potentials mainly reflect subthreshold activity. Furthermore, we showed that multisite simultaneous recordings of evoked field potentials are a valuable tool for the construction of physiological maps of the corticostriatal connections. Finally, we revealed that *in vivo* local GABAergic neurotransmission strongly modulates the temporal processing of cortical inputs.

### Evoked local field potential significance

Correlation studies between action potential discharges and local fields sustain the notion of the local origin and physiological significance of striatal field potentials [38], [39]. Also, the fact that striatal field activity is present in differential recording configurations and when local references are used supports its striatal origin [40]. Both approaches have limitations however. Striatal firing shows coupling to local field activity in the cerebral cortex, which is the main candidate for generating spurious local field activity in the striatum [16], [41]. Differential recordings favor the detection of highly localized rhythms over more synchronous activities that could however be local, like the striatal evoked potentials recorded here. The abolition of striatal evoked potentials after the local pharmacological blockade of glutamatergic AMPA receptors reported in this study, unequivocally demonstrates their synaptic nature and local origin. Although further studies are necessary to extend this finding to other forms of striatal field activity, the spatial progression of AMPA receptor blockade effects through the multielectrode recording sites (Figure 5) suggests that the contacts of the electrode used in the present study picked up activity from a relatively small volume of tissue. This conclusion is in line with recent findings in the visual cortex [42], [43].

Disentangling whether evoked local field potentials reflect a combination of subthreshold phenomena and spike discharges, or they merely reflect membrane potential fluctuations causal to spike discharges, proves to be more challenging. Previous *in vitro* studies of striatal corticostriatal transmission have described similar evoked field potentials to those reported here. The negative N2 wave of the *in vitro* striatal field response has been proposed as a population spike [20], [24] based on temporal correlations between spike discharges and the local field potential negative peak. Here we found a shorter latency of the negative N2 field potential compared to the peak response of multiunitary evoked action potentials. Moreover, MSNs respond with subthreshold dPSPs to cortical stimulation at current intensities that produce nearly maximal striatal local field responses. Thus, although spikes are seen in a majority of the striatal sites from which a local field response was recorded, it seems very likely that these spikes come from a tiny fraction of the neurons that are responding to the stimulus and hence having a small influence on the field response. Finally, intrastriatal infusion of the GABA_A_ receptor antagonist bicuculline increased striatal action potential responses to strong cortical stimulation (400 µA) which was not paralleled by an increase in the amplitude of striatal field potentials. In all, our data fit better with the view that striatal field recordings mainly reflect corticostriatal synaptic potentials which would enable further postsynaptic action potential discharges.

### Local circuit regulation through GABA_A_ receptors

In addition to the changes in the amplitude of striatal field potentials, blocking GABAergic neurotransmission increased the overall duration of the striatal field responses by more than 60%. This result indicates that the temporal processing of incoming information from the cortex is highly regulated by local GABAergic networks. Alterations in the temporal processing of cortical commands might have implications for basal ganglia syndromes such as dystonia or Tourette syndrome which have been related to alterations in striatal GABAergic interneurons [44], [45]. Such temporal regulation might be the result of blocking feedforward inhibition by fast spiking interneurons [15], [46], lateral inhibition by MSNs collaterals [47], [48], or both.

*In vivo*, cortical paired pulse stimulation induces a facilitation of the striatal response to the second stimulus, much like as it happens *in vitro*. *In vitro*, such facilitation is typically interpreted as an increased probability of glutamate release during the second stimulus due to the presynaptic accumulation of residual Ca^2+^ [30], [37]. *In vivo*, it has been argued that paired pulse stimulation reduces the discharge probability of feedforward inhibitory networks leading to a facilitation of MSNs response during the second stimulus [15]. In our study, the pharmacological blockade of GABA_A_ receptors only produced a small reduction of paired pulse facilitation, supporting the idea that it might be the consequence of a short term increase of excitatory neurotransmission.

In contrast to this small effect on paired pulse facilitation, bicuculline had a more marked effect on the duration of the paired response than on the first one. Under bicuculline infusion, the striatal field response evoked by the second stimulus was extended to a greater extent than the first one, whereas under baseline conditions, the duration of the first and second field potential responses was indistinguishable. This suggests that, under normal conditions, the amount of inhibition used to control the increased excitatory drive during the second cortical stimulus is higher than that used during the first stimulus. If fast spiking interneuron circuits are depressed at the arrival of the second stimulation pulse [15], it is likely that lateral inhibition by MSN collaterals is responsible for balancing excitation and inhibition during repetitive cortical stimulation.

Bicuculline has been shown to inhibit potassium channels of the SK family in hippocampal neurons [49]. Although we cannot completely rule out a contribution of changes in the SK current on the results reported here, our findings with bicuculline reproduce those of Pennartz and collaborators [36], who have studied the effect of picrotoxin on striatal field responses in slices.

### Corticostriatal synaptic connectivity maps

Behavioral specializations of different regions of the striatum have been well documented [4], [5]. For instance, the corticostriatal cognitive circuit involving the dorsomedial region of the striatum is thought to be related to goal directed behaviors, whereas the motor circuit involving the dorsolateral region would be concerned with habit formation and compulsive drug taking [50], [51], [52]. Moreover, individual differences in personality traits involving “reward dependence” may be accounted for by differences in the strength of anatomical connections between the prefrontal cortex and striatum [53]. In the present study we have been able to build a functional corticostriatal synaptic map originated at the prelimbic region of the mPFC which could allow measuring the strength and spatial extent of prefronto-striatal connections under different physiological and pathological conditions.

Striatal responses to prelimbic cortex stimulation were widespread but clearly regionalized displaying a maximum in the centromedial region of the dorsal striatum. The fact that a complete pharmacological blockade of field responses in some striatal sites does not change the evoked field response in spots a few hundred micrometers apart shows that field responses do not reflect volume conduction from neighboring striatal sites but biological activity of the surrounding tissue at each recording site. Furthermore, the maximal spatial extension of the evoked field response is reached with relatively low stimulation intensities and does not change during the blockade of GABA_A_ receptors, suggesting that the physiological maps truly represent the striatal area under the influence of the cortical stimulation electrode. Finally, the anatomical distribution of the corticostriatal terminals originated at the mPFC [23] is consistent with the physiological map obtained in the present study suggesting that evoked fields could be used to build high resolution physiological corticostriatal maps. Preliminary results indicate that cortical stimulation at other cortical sites map differently in the striatum in consonance with their anatomical connections.

In conclusion, the present study indicates that evoked field potentials are an adequate tool for studying corticostriatal communication *in vivo*. Taking into account the highly local nature of evoked field potentials, stimulating cortical projections belonging to different corticostriatal channels may allow studying the interface of the parallel corticostriatal circuits and striatal integration of cortical information. The relevance of this possibility is heightened when considering that basal ganglia dependent learning requires interaction among functionally distinct corticostriatal circuits and the issue of how information is transferred among the circuits still remains unanswered [4]. Furthermore, local circuit modulation by dopamine and other neuromodulators and corticostriatal synaptic plasticity might be studied *in vivo* together with basal ganglia dependent learning narrowing the gap between behavior and physiology.
